# Predicting the impact of online news articles – is information necessary? Application to COVID-19 articles

**DOI:** 10.1007/s11042-021-11621-5

**Published:** 2022-01-08

**Authors:** Judita Preiss

**Affiliations:** School of Science, Engineering and Environment, University of Salford, Salford, M5 4WT UK

**Keywords:** Twitter, Popularity prediction, Grammatical relations, SemRep relations

## Abstract

We exploit the Twitter platform to create a dataset of news articles derived from tweets concerning COVID-19, and use the associated tweets to define a number of popularity measures. The focus on (potentially) biomedical news articles allows the quantity of biomedically valid information (as extracted by biomedical relation extraction) to be included in the list of explored features. Aside from forming part of a systematic correlation exploration, the features – ranging from the semantic relations through readability measures to the article’s digital content – are used within a number of machine learning classifier and regression algorithms. Unsurprisingly, the results support that for more complex articles (as determined by a readability measure) more sophisticated syntactic structure may be expected. A weak correlation is found with information within an article suggesting that other factors, such as numbers of videos, have a notable impact on the popularity of a news article. The best popularity prediction performance is obtained using a random forest machine learning algorithm, and the feature describing the quantity of biomedical information is in the top 3 most important features in almost a third of the experiments performed. Additionally, this feature is found to be more valuable than the widely used named entity recognition.

## Introduction

Online news have become the most common source of information for many people [35], and measures of a news article’s popularity can be derived from the number of visits to a specific URL (measured by its news source) [29], or more external measures such as the number of likes, comments or shares on social networks [41]. However, it became clear during the COVID-19 pandemic that the reasons behind one news subject becoming widely popularised while another was not were not clear: hydroxychloroquine rapidly escalated to being labelled a miracle drug for battling COVID-19 in March 2020, despite very little evidence supporting its effectiveness and almost no testing of efficacy on the virus at the time, while other treatments at the same stage of trials did not gain such widespread popularity. We exploit the (potentially) medical nature of COVID-19 related news articles to investigate whether including medical based information has a bearing on the article’s resulting popularity.

While the impact of well structured documents, for example academic publications, has been evaluated retrospectively – such as within the UK’s 2014 Research Exercise Framework (REF) where 15 metrics were employed within a manual expert evaluation of almost 150,000 academic publications – they crucially employed the, post event, metric of citation counts. Potential future impact requires different approaches based on available (not future) values. Predictions regarding future scientific success have been attempted based on information contained on a scientist’s CV [1], but this approach is difficult to adapt to individual news articles written by (potentially) new journalists.

Although formal citations of a news article rarely represent its popularity, the Twitter social media platform has gained a reputation for being *the* social media platform for news [41], and therefore measures derivable from it, such as numbers of re-tweets, have been used to gauge a news article’s readership. The Twitter platform has also been used for deliberate (automatic) influence operations [2], suggesting that features can be derived to determine articles to target. News tweets, which we consider to be tweets containing a URL, have been the focus of a variety of systems, including popularity prediction (e.g. [39]), and their foundation usually lies in a number of extracted features. The main categories of features are: (i) *content features*, which aside from topic and headline information include tweet and textual elements such as hashtags [6], URLs [23] or readability [33], (ii) *user features* including followers and followees [34], and (iii) *context features* such as temporal and location aspects [31].^1^

Popularity prediction is usually carried out using machine learning: the 2015 mashable.com UCI Machine Learning Repository Online News Popularity Data Set [18] has been used extensively to predict article popularity. However, while a large number of features has undergone exploration within these systems, to our knowledge ours is the first work investigating the effect of (biomedical) information (identified by automatic relation extraction) within news articles. It is also the first work we are aware of that applies popularity prediction to news tweets regarding the COVID-19 pandemic.

The paper is structured as follows: Section 2 presents additional related work, while Section 3 outlines the technique used to create the dataset and the algorithms employed in this work. Experiments and their results are discussed in Sections 4 with 5 drawing the conclusions.

## Related work

Deciding an article’s newsworthiness from a journalist’s point of view is dependent on a number of factors. Boukes et al. [9] discuss the functional and causal models governing this, with both models relying on the concept of news factors whose presence (and higher quantity) increases a news article’s prominence. They identify seven news factors: (i) negativity (reporting of negative aspects or damage), (ii) continuity (previous mentions in the news), (iii) proximity (geographic, cultural or economic), (iv) eliteness (presence of certain individuals, organizations or nations), (v) influence and relevance (the impact of an event’s consequences), (vi) personification (reports from directly affected individuals) and (vii) conflict (confrontation and / or controversy).

As stated earlier, automatic approaches frequently focus on analysing an article’s popularity using features which are only available after publication [50]. In predictions prior to publication, which allow for improvements to the article to be made prior to release, feature sets – directly related to the above news factors – and machine learning approaches have undergone numerous refinements:

Hensinger et al. [29] use support vector machines (SVM) to predict the popularity (defined as appearing in “top stories” and “most popular” feeds) of a news article based on an extended set of features, which include a bag of word representation of the text and time related features. Moving to tweets, Petrovic et al. [42] perform a binary classification task using tweet content (hashtags, mentions etc) and user based (followers, friends etc) features to predict whether a tweet will be re-tweeted or not. Working with a corpus closest to our dataset, Bandari et al. [6] divide the number of times a news URL is posted or shared on Twitter into three categories indicating a news article’s popularity. They employ four types of features, (i) the news source, (ii) the category of news article, (iii) the subjectivity of the language in the article, and (iv) named entities, and find Naive Bayes and bagging algorithms the best performing.

Feature sets have also been further extended to include digital media content, popularity of any news articles mentioned within the publication, shares of keywords prior to publication, title polarity and LDA topics [7], and employed within a number of machine learning approaches such as random forests or adaptive boosting [18]. The latter work also gave rise to the 2015 Online News Popularity corpus, a benchmark corpus based on news appearing on mashable.com during the course of two years.^2^ Ren and Yang [45] augment the features by adding mutual information and using Fisher criterion for feature selection to achieve a performance improvement using random forests. Instead of augmenting features, feature refinements were also possible: e.g. Choudhary et al. [12] selected optimal features to employ within a Naive Bayes classifier using a genetic algorithm.

None of the works explore the importance of extractable information within the news articles as a feature. Therefore, the contributions of our work are: (1) the construction of a dataset of news articles associated with tweets regarding COVID-19, (2) the extraction of features based on information (represented by extractable relations), (3) an investigation of the importance of the information features within machine learning algorithms used to predict Twitter based popularity, and (4) an investigation of dataset balancing approaches in machine learning predictions of popularity.

## Methodology

### Dataset

To investigate the question whether the inclusion of information is required for a news article to become popular (measured using metrics based on Twitter), a dataset containing a set of news articles alongside a list of tweets mentioning these is required. Given the availability of biomedical domain tools, the probability of detectable information appearing in the news articles was increased by restricting the topic to COVID-19. While a dataset containing both (news and tweet) sets of information is – to our knowledge – not publicly available, a large number of datasets listing COVID-19 related tweets have been released. This includes Panacea lab’s COVID-19 Twitter chatter dataset for scientific use [5] which contains tweets gathered by the Twitter API using specific keywords regarding COVID-19. In August 2021, the approach was said to yield 4.4 million tweets a day, and therefore would be out of the scope of an individual researcher to gather.

Due to Twitter restrictions only tweet IDs are provided, but these can be re-hydrated to give access to the tweets’ full content and thus any URLs mentioned in the tweets can be extracted. After expanding shortened URLs (e.g. bit.ly) and following any redirects, all domains (such as reuters.com) which appeared more than 10 times were manually examined and those corresponding to a news source were retained. 15,000 URLs with a news source domain name were chosen at random and text content was extracted from their corresponding HTML pages (with information such as menus or links to other articles removed in as far as possible using simple HTML parsing tools). Any pages with no resulting content were discarded^3^ as were web pages in languages other than English (identified using Perl’s Lingua::Ident, language identification software based on [16]) and URLs which failed retrieval. This gave rise to 12,488 distinct URLs with content, arising from 476 distinct news sources.

**Table 1:** Overview of news article based features

Feature category	Specific features
Text based	Number of words in the title & body of text, average word lengths in title & body of text, total number of sentences in title & body of text, number of words per sentence, numbers of images and videos.
Readability based	Readability measures as described in Table 2, computed by the textstat and readability python packages.
Semantic content based	Numbers of grammatical relations extracted by a parser tuned for biomedical text (SemRep) [46] and a generic grammatical relation extraction parser (Stanford) [36], and the quantity of named entities contained in the text determined by Stanford NER [20].

### Feature extraction

The second step involves the extraction of features from a news article. These can be divided into three categories as described below (see Table 1 for a summary). Note that the domain and journalist name are explicitly excluded as predictions are based on the content of the article alone to allow for previously unseen news sources.

#### Textual information

The numbers of images and videos within the textual content of each news article were noted. Other directly computable information was extracted from the text: this ranges from simple information regarding an article’s length, number of sentences in the text, through average number of characters per word to more syntactic based information such as the usage of “to be” verbs, pronoun counts (potentially suggesting personification) or the average number of sentences starting with a pronoun.

A number of these factors can be combined into a single value describing an article’s readability – its appropriateness for audiences at various stages of education. A number of readability measures have been widely used to evaluate a text’s suitability for its target audience. The measures employed in this work and the information they are based on, along with the measure’s original purpose, are listed in Table 2. The calculations range from simple weighted combinations based on numbers of characters, words and sentences (e.g. ARI = $4.71 \frac{\text{characters}}{\text{words}} + 0.5 \frac{\text{words}}{\text{sentences}} - 21.43$) to complex calculations (e.g. SMOG = $1.0430\sqrt{\text{polysyllables} \ast \frac{30}{\text{sentences}}} + 3.1291$). Two implementations of these measures are used, Python’s textstat and readability, as there are differences in their implementations and it is not clear, without investigation, which would be more suitable.

**Table 2:** Summary of readability measures

Readability measure	Purpose	Based on numbers of ...
Flesch Reading Ease (FRE) and FleschKincaid Grade Level (F-K) [21]	General	Syllables, words and sentences.
Automated readability index (ARI) [48]	Technical	Characters, words and sentences.
Coleman-Liau (C-L) [13]	Education	Characters, words and sentences.
Gunning Fog Index (FOG) [25]	Business & product	Words, complex words ($>3$ syllables) and sentences.
Simple Measure of Gobbledygook (SMOG) index [37]	Healthcare	Sentences and polysyllabic words.
Dale-Chall index (D-C) [15]	General / education	Words, sentences and ‘difficult words’ from own set.
Linsear Write metric (LW) [40]	Technical	Easy and hard words, sentences.

#### Syntactic and semantic information

The question under investigation is whether the presence or absence of (automatically extractable) information has an effect on a news article’s popularity. Features representing the quantity of information in an article therefore also need to be included. Based on work in literature based discovery [43], where information is extracted from publications using grammatical relation triples, and the importance of named entities in news articles (e.g. [24]), three additional pieces of information are extracted:

**Stanford grammatical relations** The publicly available Stanford probabilistic lexicalized dependency parser extracts grammatical relations (GRs) from sentences [36]. Such GRs are triples consisting of the name of the relation, the governor and the dependent. For example, for the sentence

According to the WHO, the most common symptoms of Covid-19 are fever, tiredness and a dry cough.

For this sentence, the extracted Stanford GRs include:^4^
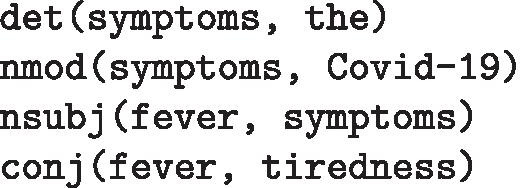


**SemRep relations** Since the dataset is restricted to COVID-19, the biomedical tool SemRep [46] can be used to extract semantic predications. Similarly to the Stanford GRs, these consist of a subject argument, an object argument and a binding relation. However, unlike the Stanford GRs, the arguments must appear in the Unified Medical Language System (UMLS) metathesaurus [8] and the relation is constrained to those appearing in the UMLS semantic network. The above sentence yields the following SemRep relations:
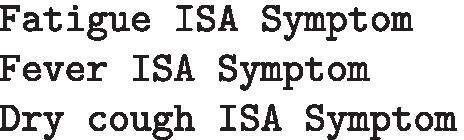


**Stanford named entities** Since mentions of certain people, organizations or locations are known to increase an article’s newsworthiness, named entities (NEs) are extracted using the Stanford named entity recogniser [20]. This is a conditional random field classifier which identifies person, organization and location entities in English text. In the running example, the NE system identifies *WHO* to be a named entity (ORGANIZATION).

### Measures of popularity

Since the dataset contains news articles linked with tweets, the associated tweet metadata can be used to assign a popularity value to each news article. The following options for defining the popularity measure are explored:

1. The average number of re-tweets. The reposting of another user’s tweet is referred to as re-tweeting. If a particular tweet containing a URL has a high re-tweet count, it is hypothesized that the web page contained within is highly influential. The normalized sum of re-tweets of all tweets containing a specific URL yields a URL per tweet value of *num_retweets*.
2. A combination of re-tweets and followers. Each Twitter user has some number of followers, meaning that not all re-tweets are equal – a single re-tweet by a user with 1,000,000 followers will reach more people than 1,000 re-tweets by people with 10 followers each. For a single URL, the number of followers of all users who either wrote a tweet containing this URL or re-tweeted such a tweet are combined to give *num_followers*. Given the extremes of this measure (while many users have relatively low numbers of followers, some users have extremely high counts), a log version of this measure is also explored.
3. The number of favourites beside a tweet. Each user has the option to place a favourite / like alongside a tweet, and the overall number of these may serve as an indicator of the size of the audience the tweet has reached.
4. Average number of hashtags. These single word or phrase expressions attached to tweets link tweets with the same hashtag together, potentially reaching a larger audience via this categorization. Multiple tweets of the same URL, tweeted by different users, can be tagged with different numbers of hashtags, therefore an average number of hashtags per number of tweets containing a given URL is considered.

While the problem of spread of fake news, in particular using Twitter bots (e.g. [49]), does not directly impact this work (as mentioned in Antenore et al. [4], when detecting popularity of a piece of news, a bot in Twitter is effectively regarded as a credible source of information, counting the same as a human user), it needs to be mentioned. For example, Wojcik et al. [51] suggest that 66% of tweeted links to popular websites are due to bots, however Ferrara [19] shows that the focus of automatic tweets appears to be more frequently political than health based. This is supported by a brief exploration of the dataset in this work: the probability of being a bot account, measured by Botometer [47], was evaluated for 2500 randomly selected Twitter accounts from the list of tweeters / re-tweeters / followers appearing in the dataset. A threshold of 0.76, as used in [32], suggests 28% bots, which is significantly lower than the 53%-66% expected bots tweeting about COVID-19 [30]. The difference is believed to be due to the specific dataset being used.

### Correlation measures

The relationship between a news article feature and the article’s popularity (as defined above) can be analysed using correlation measures. The following correlation measures are explored in this work:

**Pearson correlation coefficient** The Pearson correlation coefficient (r) measures the linear correlation between two variables using the formula

$$ \frac{cov(X,Y)}{\sigma _X \sigma _Y} $$

where *cov* is the covariance and $\sigma$ the standard deviation. It has a value between -1 and 1 with 1 representing a total positive correlation. The correlation also returns a *p*-value, the probability that the same result would have been observed if the correlation coefficient was zero. A result is deemed statistically significant when $p < 0.05$.

**Spearman’s rank** The Spearman’s rank correlation coefficient ($\rho$) investigates how well a monotonic function can represent the relationship between two variables. Instead of operating directly on the raw variable values *X*, it converts these to ranks (i.e. relative position within the variable, 1st, 2nd etc) $rg_X$. The formula is then the Pearson correlation formula applied to ranks

$$ \frac{cov(rg_X, rg_Y)}{\sigma _X, \sigma _Y} $$

A value close to 1 represents similar rank distributions while -1 indicates dissimilarity.

**Kendall rank** For a set of observations $(x_1, y_1), \cdots , (x_n, y_n)$, the Kendall rank correlation coefficient ($\tau$) relies on the number of concordant (i.e. pairs where for $(x_i, x_j)$ and $(y_i, y_j)$ with $i < j$, either $x_i > x_j$ and $y_i > y_j$ or $x_i < x_j$ and $y_i < y_j$) and discordant (otherwise) pairs:

$$ \frac{\text{(num concordant pairs)} - (\text {num discordant pairs})}{{\left( \begin{array}{l}n \\ 2\end{array}\right) }} $$

### Machine learning algorithms

The hypothesis that the popularity of an article can be predicted based on derivable features is explored using a number of machine learning algorithms. Since the popularity measures (defined in Section 3.3) yield continuous, numerical, values, the problem can be framed either as a (binary) classification task, based on a threshold, or a regression problem. Regression predicts the expected popularity measure value, such as the number of re-tweets, while for classification, the popularity measure is converted into two classes, making the predictions a binary choice, *influential* or *not*, for each news article. The following machine learning algorithms are explored:

**Decision tree** The decision tree (DT) algorithm constructs a structure of nodes connected in a tree-like pattern, where each node split corresponds to a condition on a feature variable which best splits the training data (e.g. [44]). DTs can be used for either classification (DTC) or regression (DTR). A new instance is classified by following the path of its feature values down the tree until a leaf node is reached.

**Random forest** Random forests build a number of DTs from random samples, and subsets of features, of the training data (e.g. [10]). A new instance is classified by following all the trees to their leaves and either outputting the majority class (classification, RFC) or an average of the individual trees (regression, RFR).

**Gradient boosting** Gradient boosting (GBC) also builds multiple DTs, however – unlike random forests which build DTs independently – this approach builds its DTs one at a time, such that the new DT compensates for the shortcomings of the previous DTs (e.g. [22]). There is therefore no need to combine results of multiple DTs when classifying, as precisely one result will be reached when the trees are followed.

*k***-nearest neighbours (KNN)** All training feature vectors are stored and, when used as a classifier, a new instance is classified based on the most common class among the new instance feature vector’s *k* closest training instances (e.g. [3]).

**Support vector machines (SVM)** For a binary classification problem, the training feature vectors are mapped to a (high dimensional) space where a hyperplane separating the classes can be found (e.g. [14]). New instances are classified by applying the same mapping and finding which side of the maximum-margin hyperplane the new instance lies.

**Multilayer perceptron** The multilayer perceptron (MLP) is a neural network with at least one hidden layer which employs (potentially non linear) activation functions and uses backpropagation for training (e.g. [27]). In binary classification, a trained MLP is used to classify a new instance and yield the predicted value directly.

#### Hyperparameter tuning

Each machine learning algorithm has parameters (such as the value of *k* in *k*-nearest neighbours) which need to be tuned. Hyperparameter tuning is performed using python’s GridSearchCV with a 0.1 validation split on the training portion of the dataset (80% of the data) and 10 cross validation folds. Details of the parameter grids explored for each algorithm are included in Appendix 1. Overall performance of the tuned system is evaluated on a (separate) test corpus using precision, recall and F-measure ($F_1$).

### Balancing the dataset

When the problem is treated as binary classification, the training data may contain unequal portions of the two classes: since only a small proportion of news articles are influential, a smaller portion of the training data is expected to belong to the *influential* class. A number of techniques for addressing the problem of data imbalance exist and the following are explored:

**Random undersampling** Samples are randomly selected from the majority class and removed from the training set, until the desired balance is reached.

**Random oversampling** Samples from the minority class are randomly repeated in the training set, until the desired balance is reached.

**Synthetic minority oversampling technique (SMOTE)** This approach adds synthetically created minority class examples to the training data. These are created by focusing on a random example from the minority class, *A*, and selecting one of its *k* nearest minority class neighbours at random, *B*, and constructing a new instance between *A* and *B* in the space [11].

**SMOTE + undersampling** The first step involves undersampling of the majority class while the second employs SMOTE to boost the number of minority class examples.

**Borderline SMOTE** This algorithm uses a KNN model to identify misclassified examples and oversamples just these, difficult, instances [26].

**SVM SMOTE** The misclassified examples used to generate synthetic examples are based on an SVM instead of a KNN in this instance [38].

**Adaptive synthetic sampling (ADASYN)** The examples to oversample are chosen based on the number of majority class examples in the KNN neighbourhood of each minority example, adding the largest number of minority examples where there are fewest in the original training data [28].

## Experiments and results

An initial exploration of correlations between the features extracted in Section 3.2 and the popularity measures from Section 3.3 was performed. While a strong direct correlation was not expected – a single feature is unlikely to be the only factor in determining an article’s influence – the correlations may provide a ranking of the features which can be later compared to those found important by the machine learning algorithms.

**Table 3:** Correlations between the number of re-tweets and some of the features

Feature	Restriction	Pearson		Spearman		Kendal	
		r	p	$\rho$	p	$\tau$	p
Avg Stanford GRs	SMOG $> 16$	0.38	0.00	0.21	0.07	0.18	0.05
Avg SemRep GRs	ARI $> 17$	0.12	0.15	0.27	0.00	0.22	0.00
Number of videos	ARI $15 < x \le 16$	0.45	0.00	0.28	0.00	0.25	0.00
Avg word length in title	D-C $< 5$	−0.8	0.00	−0.32	0.19	−0.26	0.17

### Correlation results

As expected, computing correlations across the entire news web page dataset did not yield any strong correlations. This is even less surprising when the range of readability values over the collection are taken into account: for example, the Sun newspaper is said to have FRE of about 64, the Time magazine about 52 and the Harvard Law Review scores around 30. Documents with vastly different readability values can be expected to have very different content and readership, and therefore vary in popularity measures.

A second set of correlations is therefore computed between subsets of the entire news web page dataset corresponding to various readability ranges and popularity measures. Table 3 presents a selection of these results with the “restriction” column refercle D-C $5 \leq x < 6$ represents URLs with Dale-Chall index in the range 5-6 which corresponds to texts easily understood by 5th or 6th grade students. This time, some correlations are apparent: for example, an unsurprising moderate correlation can be seen between documents with ARI between 15 and 16, i.e. 10th grade accessible, and the number of videos in the document.

Lastly, correlations of popularity measures and the quantity of information in the document, as represented by Stanford and SemRep relations, are explored. When computing correlations between the number of Stanford grammatical relations per sentence in an article against the number of re-tweets, Pearson’s r finds a moderate correlation for documents with SMOG (i.e. grade level) $>16$, and Spearman and Kendal indicates a weak correlation for this case. Similar correlation computed with SemRep relations results in a weak Spearman and Kendal correlation in documents with ARI $> 17$ (12th grade and above). However, the overall number of SemRep relations in the collection is low – SemRep is tuned to the biomedical domain, only capable of extracting relations between UMLS concepts – and the result can be assumed to be influenced by a large number of zero SemRep relation counts. Surprisingly, contrary to the expected importance of named entities mentioned previously (and their presence in related works), no correlations were found between the number of re-tweets and the number of NEs found.

### Statistical analysis of the popularity measures

Statistical analysis can be used to guide the selection of the popularity measure: this can be seen in Table 4. In all cases standard deviation exceeds the mean, indicating that the data is not normally distributed. Median exceeding the mean suggests a positive skew to the distribution, which is supported by the high maximum values. With the possible exception of log(followers), also included in table, experimentation did not indicate a log-normal distribution for the remaining popularity measures.

**Table 4:** Statistical overview of features considered as popularity measures

	Favourites	Followers	Hashtags	Re-tweets	Log_followers
Minimum	0.0	0.0	0.0	0.0	0.0
1st quartile	0.0	306.5	0.0	0.0	5.7
Median	0.17	1973.0	0.0	0.0	7.6
3rd quartile	1.0	17038.0	1.0	2.0	9.7
Maximum	1342.0	26200902	19	17779.4	17.1
Mean	2.28	219892.0	0.7	9.7	7.9
Standard deviation	26.7	1329625.9	1.6	2480996.8	3.1

**Fig. 1:**
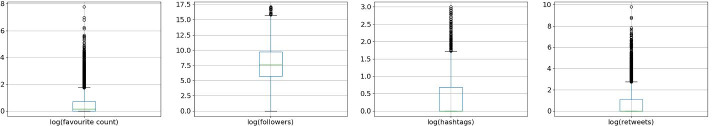
Boxplots for log(distribution) of the four importance features: favourite count, followers, hashtags, re-tweets

The log distributions are easier to examine using a boxplot (see Fig. 1): for a normal distribution, the mean should appear central to the box drawn between first and third quantiles with symmetric whiskers and few outliers. This shape is only evident for log(followers), however a slight doubt is cast by an atypical number of outliers.

### Predicting the popularity measure: classification threshold

**Fig. 2:**
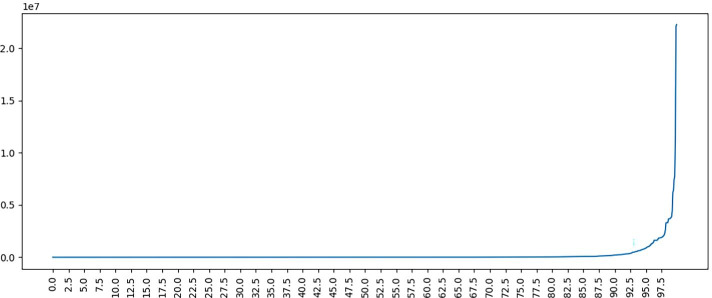
Quantile plot for *followers*

While the popularity measure value can be predicted directly, for example using regression, the task can also be set up as binary classification. To this end, a threshold needs to be chosen for the popularity value to divide the training data into two classes. The selection of an optimal value of the threshold can be guided by a plot of quantiles: for example the maximum number of followers at 10%, 15%, 20%, ...of the data indicates a steady rise until the final sharp rise (shown in Fig. 2). This shape is consistent for all four popularity measures investigated, suggesting that a suitable division of popularity is likely in the final section after the gradient change. The number of resulting *non influential* and *influential* articles corresponding to thresholds of 85%, 87.5% and 90% can be seen in Table 5. For example, for the 85% threshold, news articles appearing in tweets with fewer than 4.1 average re-tweets will be deemed non influential, while ones exceeding this number will be considered influential.

**Table 5:** For each percentage division, the actual value is given and the number of non influential (0) and influential (1) articles this yields

	85%			87.5%			90%		
	value	0	1	value	0	1	value	0	1
Favourites	2	10432	2056	2.4	10927	1561	3	11142	1346
Followers	79902	10517	1971	114611	10927	1561	214131	11233	1255
Hashtags	1.5	10609	1879	2	10841	1647	2	10841	1647
Re-tweets	4.1	10477	2011	5.5	10927	1561	7.7	11239	1249

#### Machine learning predictions and discussion

**Fig. 3:**
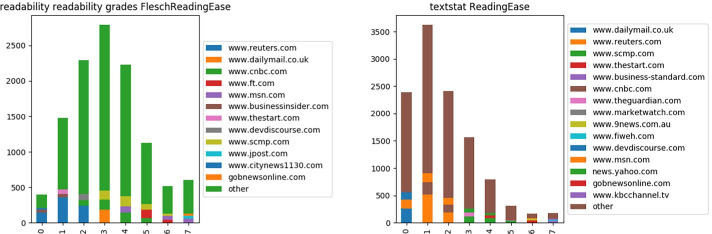
Distribution of most common sources in Flesch Reading Ease; readability calculation on left, textstat on right

The features described in Section 3.2 give rise to a vector of length 74 for each URL included in the dataset. However some features – particularly the readability grades produced by two different python implementations – may be believed to be duplicates. Further exploration shows that the two implementations often differ in the values they assign: see Fig. 3 which shows the distribution of Flesch Reading Ease values as assigned by the two algorithms to the same set of news articles resulting in two non-identical distributions with differences likely due to their definitions of a word and a syllable. Feature sets reduced to one or the other implementation, as well as only the readability grades outputs of the readability implementation (rather than the sentence information, word usage and sentence beginnings features also produced by this implementation) were explored. See Table 6 for information regarding the corresponding number of features remaining after a restriction is applied.

**Table 6:** Feature vector lengths when restrictions are applied

Restriction	Readability grades	Textstat	Readability	All
Num features	24	38	50	74

**Fig. 4:**
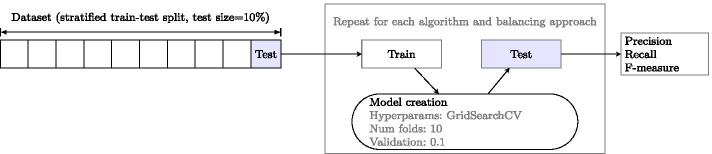
Experiment setup

The experimental setup for the optimization of feature set, classification threshold, ML algorithm (with hyperparameters) and approaches to data imbalance, partly outlined in Section 3.5.1, can be seen in Fig. 4: to ensure validity of results, hyperparameter optimizations are carried out on the training split of the data using 10 fold cross validation. The best results were obtained using the 50 readability features and the followers popularity measure. The results of the best algorithm, optimal hyperparameters and method for addressing dataset imbalance for each threshold are presented in Table 7 with the best results for each algorithm available in Appendix 2. Overall, random forests appear to be best suited to the problem, as has been the case in previous works [18, 45], however the differences between the top approaches appear less significant with an increased threshold value – the top 4 approaches are within 0.01 F-measure for 90% threshold. This, along with an overall decrease in F-measure achieved with an increased threshold, is not unexpected: the problem becomes more difficult as the number of popular instances in the dataset decreases – for a start, the quantity of popular instances in the training data also goes down.

**Table 7:** Best performing (F-measure, $F_1$) combination of algorithm and dataset balancing for each threshold (T) using the readability features

T	Algorithm & optimal hyperparameters	Balancing	$F_1$
80%	RFC	Random oversampling	0.504
	criterion: gini, max_depth: 23		
	max_features: log2, n_estimators: 500		
82.5%	RFR	Random oversampling	0.482
	criterion: mse, max_depth: 16		
	max_features: auto, n_estimators: 200		
85%	RFC	Borderline SMOTE SVM	0.438
	criterion: gini, max_depth: 15		
	max_features: auto, n_estimators: 800		
87.5%	RFC	Borderline SMOTE SVM	0.361
	criterion: gini, max_depth: 15		
	max_features: sqrt, n_estimators: 800		
90%	RFC	SMOTE & undersampling	0.338
	criterion: entropy, max_depth: 23		
	max_features: sqrt, n_estimators: 1100		

It is unclear whether F-measure is the optimal evaluation measure as a very high precision can be achieved at the expense of a lower recall: for example, the RFC algorithm with no balancing attains 0.923 precision (with 0.192 recall) on the 80% threshold dataset. This means that a number of news stories can be labelled as popular by the system with very high accuracy – if the system is used to recommend articles for revision in order for them to reach high popularity, the aim would be for the revised article to become a member of this, easily detectable, group.

Focusing on the information encoding features, since SemRep relations are (a) specific to the biomedical domain, and (b) employ a restricted set of binding relations, they are expected to be more useful in encoding information for the purposes of this study. This hypothesis is supported by an exploration of the feature importances yielded by the best performing RFC algorithms, for example the top 3 features for the best algorithm using a threshold 80% are: (1) number of images, (2) *SemRep relations*, (3) sentences beginning with conjunction. Table 8 shows the rank of the relation features over all combinations of dataset balancing and all thresholds (across the RFC and DTC algorithms which produce feature rankings). 50 features were explored and the number of SemRep relations was used as the initial split 22% of the time (half of the time in RFC and half in DTC). “Average SemRep” divides the total number of SemRep relations in the article across the tweets that directly tweeted the URL, yielding a very small number as indicated by the last columns describing the mean and standard deviation on the 80% threshold dataset. On the other hand, the high number of Stanford relations present in articles over all makes an average more useful, though not as useful as named entities (which have been used in popularity predicting in the past).

**Table 8:** Statistical information regarding information containing features for threshold binary80

Relation	Feature rank				80%	
	1	1-3	1-5	1-10	Mean	SD
Semrep	22%	31%	33%	34%	2.95	6.68
Average semrep	0%	0%	1%	19%	0.03	0.05
Stanford	0%	0%	0%	0%	1322.48	2095.63
Average stanford	0%	0%	2%	33%	12.68	4.98
Named entity	0%	3%	8%	19%	72.28	140.05

The MLP algorithm did not perform as well as expected and it is believed that a much larger training set is required for this approach. While the ability to acquire a larger training dataset is restricted by the manual component (such as the identification of news URLs) and limits imposed by Twitter, it is believed that a larger dataset may also compensate for the noise present in the dataset: aside from information (such as user accounts) being removed from Twitter, follower counts may be being affected by exceptional cases, e.g. a tweet being re-tweeted by a user with a follower count of 1M+. Despite the possible further gains available from a larger dataset, we have shown that predictions can be made regarding a COVID-19 concerning news article’s popularity based on features extracted from the article prior to its publication and that features based on information encoding relations (such as SemRep) are ranked higher in importance than previously regularly exploited named entities.

## Conclusion

An investigation was performed into the suitability of features based on relation information for predicting (Twitter based) popularity of news articles. The number of Stanford grammatical relations was found to have a moderate correlation with the number of re-tweets for complex articles, while SemRep relations – semantic relations pertinent to the topic under investigation – were found to have a weak correlation for articles with reading grade 12 and above. When using a popularity metric based on a combination of followers and re-tweets, the highest performance was achieved by random forest algorithms and, across random forest and decision tree algorithms, the SemRep relations feature ranked in the top three in just under a third of the cases explored. Moreover, these information based features ranked higher in importance than previously regularly exploited named entity based features.

The investigation was performed on COVID-19 news articles, where the presence of information – particularly that derivable by the biomedical relation extraction tool SemRep – was expected. It is not clear whether the same result would be expected in another domain, as, for example Fazel and Wolf [17] showed that there was no correlation between an article with a top REF score (which presumably contains a large quantity of information) and its associated Twitter activity. However, the contribution of the work in predicting the popularity of health related news articles should not be underestimated.

## Appendix A: Hyperparameter tuning

**Table:**

Algorithm	Abbrev	Parameter grid
Decision tree classifier	DTC	max_depth: range(3,20)
		criterion: [gini, entropy]
Decision tree regressor	DTR	max_depth: range(3,20)
		criterion: [mse, mae]
Random forest classifier	RFC	n_estimators: [200, 500, 800, 1100]
		max_features: [auto, sqrt, log2]
		max_depth: range(8,25)
		criterion: [gini, entropy]
Random forest regressor	RFR	n_estimators: [200, 500, 800, 1000]
		max_features: [auto, sqrt, log2]
		max_depth: range(8,25)
		criterion: [mse, mae]
Gradient boosting	GBC	learning_rate: [0.05, 0.1, 0.2, 0.5]
		n_estimators: [100, 200, 500]
		max_features: [log2, sqrt]
		max_depth: range(4,12,2)]
		criterion: [friedman_mse, mse]
*k*-nearest neighbours	KNN	n_neighbors: range(1, 31)
		weights: [uniform, distance]
Support vector machines	SVM	C: [0.1, 1, 10, 100]
		gamma: [0.1, 0.01, 0.001]
		kernel: [rbf, poly]
Multilayer perceptron	MLP	activation: [relu, tanh]
		hidden_layer_sizes: [(50,), (25,50,), (25,37,50,)]
		solver: [adam, lbfgs]
		early_stopping: [True]
		max_iter: [5000]

## Appendix B: Best results for each ML algorithm

**Table:**

Threshold	Algorithm	Balancing	F-measure
80%	RFC	Random oversampling	0.504
	RFR	Random oversampling	0.496
	SVM	Random oversampling	0.476
	KNN	ADASYN	0.459
	MLP	SMOTE	0.457
	GBC	Borderline SMOTE SVM	0.454
	DTR	Random oversampling	0.446
	DTC	Random oversampling	0.438
82.5%	RFR	Random oversampling	0.482
	RFC	SMOTE	0.475
	SVM	Borderline SMOTE SVM	0.450
	GBC	SMOTE	0.446
	KNN	Borderline SMOTE	0.428
	DTC	SMOTE & undersampling	0.415
	DTR	Random over	0.409
	MLP	ADASYN	0.409
85%	RFC	Borderline SMOTE SVM	0.438
	RFR	Random undersampling	0.433
	GBC	Borderline SMOTE	0.413
	SVM	Random undersampling	0.405
	MLP	Random undersampling	0.381
	KNN	Random undersampling	0.377
	DTC	Borderline SMOTE SVM	0.375
	DTR	ADASYN	0.358
87.5%	RFC	Borderline SMOTE SVM	0.361
	GBC	Random undersampling	0.351
	SVM	Random undersampling	0.350
	RFR	Random undersampling	0.342
	MLP	SMOTE	0.321
	KNN	Random undersampling	0.319
	DTC	Random oversampling	0.310
	DTR	Random oversampling	0.310
90%	RFC	SMOTE & undersampling	0.338
	RFR	SMOTE & undersampling	0.336
	SVM	SMOTE & undersampling	0.332
	KNN	SMOTE & undersampling	0.330
	MLP	SMOTE	0.299
	GBC	Random undersampling	0.279
	DTC	ADASYN	0.273
	DTR	Borderline SMOTE	0.270

## Footnotes

1 Note that the focus of this work on COVID-19 news articles takes away the need to encode the topic and location aspects.

2 Note that while this dataset could be thought to be perfect for our investigation, as the news articles in this dataset are annotated with popularity, Twitter restrictions prevent access to tweets from the time of their release and some URLs can no longer be retrieved.

3 Note that such cases were most often due to the incompatibility of the page’s HTML with the parser used.

4 The position of each word in the input is also included in the default Stanford GR output but this has been removed in the example for ease of reading.
